# Physiological and morphological plasticity in *Stylophora pistillata* larvae from Eilat, Israel, to shallow and mesophotic light conditions

**DOI:** 10.1016/j.isci.2023.106969

**Published:** 2023-05-26

**Authors:** Jessica Bellworthy, Rachel Pardo, Federica Scucchia, Paul Zaslansky, Gretchen Goodbody-Gringley, Tali Mass

**Affiliations:** 1 Department of Marine Biology, Leon H. Charney School of Marine Sciences, University of Haifa, Haifa, Israel; 2 Interuniversity Institute of Marine Sciences, Eilat, Israel; 3 Department for Operative and Preventive Dentistry, Charité Dental School – Charité – Universitätsmedizin Berlin, Berlin, Germany; 4 Central Caribbean Marine Institute, Little Cayman Island, Cayman Islands, UK; 5 Morris Kahn Marine Research Station, The Leon H. Charney School of Marine Sciences, University of Haifa, Sdot Yam, Israel

**Keywords:** Marine organism, Aquatic science, Zoology, Aquatic biology

## Abstract

Mesophotic reefs have been proposed as climate change refugia but are not synonymous ecosystems with shallow reefs and remain exposed to anthropogenic impacts. Planulae from the reef-building coral *Stylophora pistillata*, Gulf of Aqaba, from 5- and 45-m depth were tested *ex situ* for capacity to settle, grow, and acclimate to reciprocal light conditions. Skeletons were scanned by phase contrast-enhanced micro-CT to study morphology. Deep planulae had reduced volume, smaller diameter on settlement, and greater algal symbiont density. Light conditions did not have significant impact on settlement or mortality rates. Photosynthetic acclimation of algal symbionts was evident within 21–35 days after settlement but growth rate and polyp development were slower for individuals translocated away from their parental origin compared to controls. Though our data reveal rapid symbiont acclimation, reduced growth rates and limited capacity for skeletal modification likely limit the potential for mesophotic larvae to settle on shallow reefs.

## Highlights

• *Stylophora pistillata* coral larvae from Eilat have depth-dependent phenotypes
• Larval survival and settlement did not depend on origin or experiment light levels
• Juvenile corals grew fastest in their native parental habitat
• Algal symbiont photoacclimation to novel light conditions occurred within 35 days

## Graphical abstract

**Figure:**
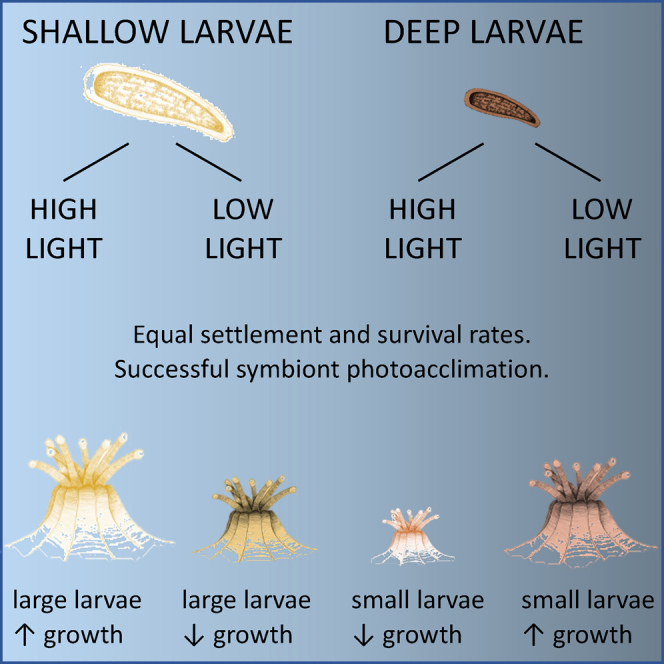

## Introduction

In the last few decades, coral reefs have been increasingly damaged by anthropogenic impacts including climate change, eutrophication, and overfishing.^1^^,^^2^^,^^3^ These impacts are often most visibly evident on shallow reefs, whereas mesophotic reefs (beyond 30 m water depth) can host relatively stable populations,^4^ in some cases with higher coral abundance and larger colonies.^5^ Consequently the optimistic Deep Reef Refuge Hypothesis emerged.^6^^,^^7^ However, deeper reefs are not universally protected from impact with recent studies documenting storm damage,^8^ plastic pollution,^8^ thermal bleaching,^9^^,^^10^ and disease^11^^,^^12^^,^^13^ at mesophotic depths. In addition, mesophotic corals often have lower productivity, growth rates, and reproductive output relative to shallow conspecifics.^14^^,^^15^^,^^16^^,^^17^ Furthermore, abiotic gradients such as light intensity, spectral range, and wave action cause depth-dependent differences in coral physiology,^18^^,^^19^ morphology,^20^^,^^21^^,^^22^ and molecular processes^22^^,^^23^ that make shallow and deep reefs unique. Reduced light intensity with increasing water depth typically results in more plate-like coral colonies, with smaller calices, greater distance between polyps, higher chlorophyll concentrations, and greater photosynthetic efficiency.^18^^,^^20^^,^^21^ Finally, though species with a wide depth range may be described as depth generalists, the depth range of peak ecological fitness and productivity may be much narrower.^24^ Together, these processes may limit the capacity of mesophotic reefs to act as coral refugia.

Part of the Deep Reef Refugia Hypothesis suggests that coral larvae from deep reefs may replenish shallow reefs following disturbance. This assumes that the reefs are physically connected (i.e., by water flow), that deep conspecifics are reproductively active, and that larvae from deep reefs are viable on shallow reefs. Like adult corals, larvae (planulae) of some coral species are noted to have depth-dependent phenotypes that closely match those of their parents, particularly species with an internal brooding mode of reproduction.^25^ This resemblance may confer higher fitness to planulae that settle within their parental environment acting as a barrier preventing cross-depth dispersal.^26^ In addition, morphological and physiological characteristics, such as lipid concentration^27^ and size^28^ of larvae have important implications for dispersal potential, which may be especially important following environmental disturbance or changes in the regional climate. If the environment becomes stressful, long-range dispersal may increase the chances that offspring will escape from stressful parental environments. However, for progeny to settle in the new environment, phenotypic plasticity is of paramount importance. Concomitantly, within the plankton, coral planulae possess little directional control and can therefore be advected to a reef with environmental conditions markedly different from their origin.^29^ Such unpredictability in settlement environment will further favor the survival of larvae with high developmental plasticity.^30^^,^^31^

Although studies performing translocation to assess adult coral acclimation are increasingly common and have demonstrated both plastic and fixed traits,^19^^,^^22^^,^^32^ only three studies to date address the same questions regarding coral planulae. Planulae of *Porites astreoides* colonies in Bermuda had higher settlement success under mesophotic light conditions (*ex situ*) irrespective of parental depth origin (10 or 45 m deep).^33^ In contrast, shallow origin planulae of *Stylophora pistillata* in Eilat settled equally well under simulated shallow and mesophotic light conditions, however planulae of the mesophotic depth specialist *Stylophora kuehlmanni* had more than double settlement rates at low light conditions compared to high light.^26^ When offered a choice between settlement tiles pre-conditioned at different depths, planulae exhibited a significant preference for tiles from their parental origin environment. In addition, when monitored *in situ* after settlement, juvenile survival rates of *S. pistillata* were low at their non-native depth relative to their origin depth. These trends are like those observed for mesophotic (40 m) *Seriatopora hystrix* from Okinawa, Japan, which displayed bleaching and significantly decreased survival rates at 5 and 10 m depth compared to juveniles at 20 and 40 m depth.^34^ This interesting mix of conclusions concerning settlement and survival probability of planulae under different light conditions warrants additional studies.

The present study sought to expand on these previous studies. We sampled planulae from the brooding reef building coral *Stylophora pistillata* in the Gulf of Aqaba, where it inhabits a relatively wide depth range of 0 to approximately 60 m^5^^,^^35^ with high phenotypic plasticity over that depth range.^18^^,^^19^^,^^22^^,^^36^ In a two-month *ex situ* experiment, we examined how depth-dependent differences in planulae phenotype influence settlement and colony establishment when planulae are exposed to a contrasting light (depth proxy) environment relative to their parental origin. Building on settlement and survival data presented in previous studies, we also examined morphology, growth, and photophysiology at multiple time points to assess the degree of acclimation toward settlement environment conditions. Synchrotron based microCT was used to visualize the coral morphology in 3D and compare it across settlement conditions. The ability to acclimate may add support to the notion that settlement across a broad vertical depth gradient is a viable hypothesis, whereas juvenile growth rates, survival, and realized competitive interactions in nature will also ultimately define whether shallow and mesophotic coral populations buffer each other.

## Results

### Larval physiology

**Figure 1:**
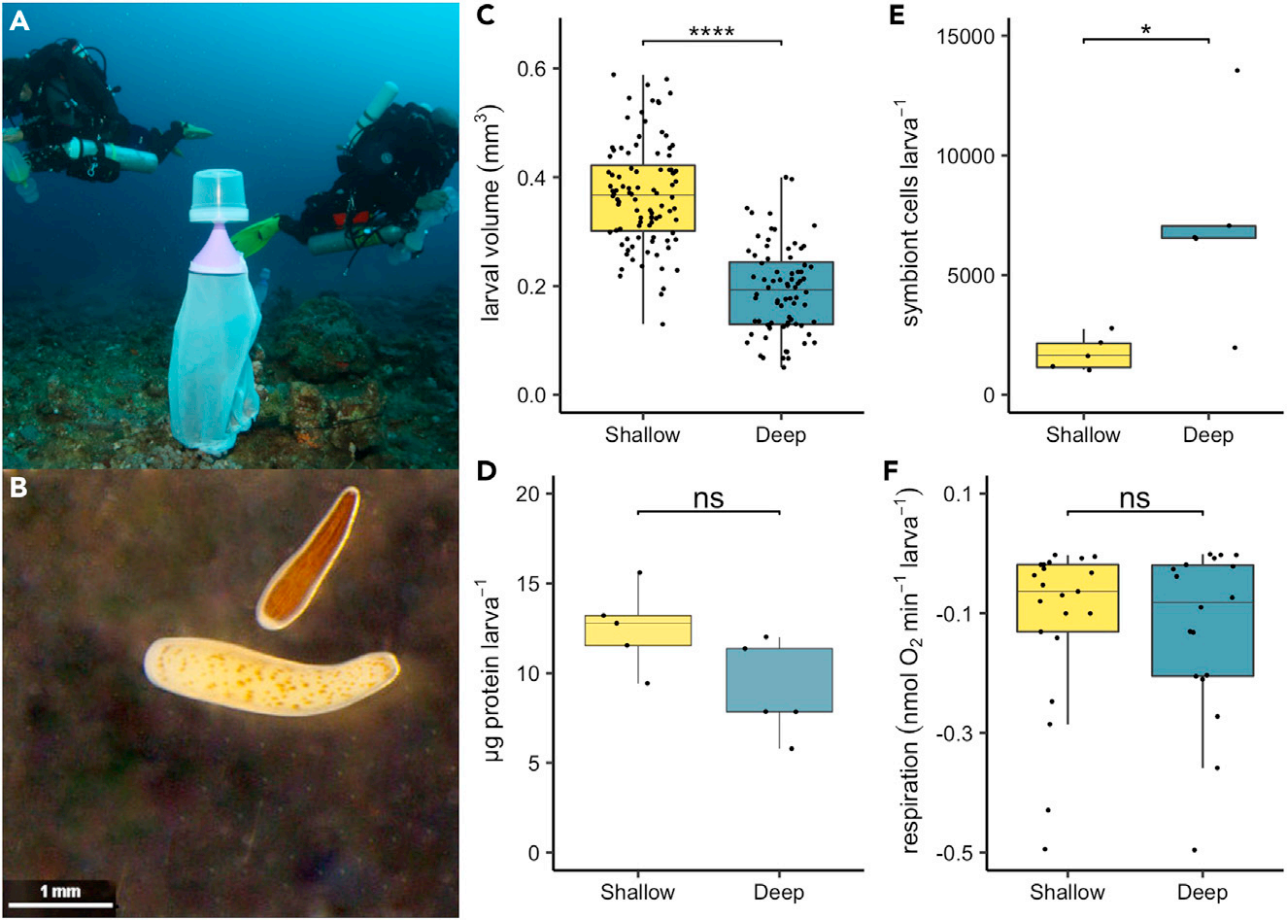
Larval physiology from shallow (yellow) versus deep (blue) colonies (A) Closed Circuit Rebreather divers setting planulae traps at 45 m depth in Eilat, photo by H. Nativ. (B) Planulae from deep (top) and shallow (bottom) colonies taken using a binocular microscope, photo by J. Bellworthy. Note differences in pigmentation and size. (C) Elliptical volume of planulae (n shallow = 96, n deep = 76). (D) Protein concentration per individual planula (n = 5 replicates per depth). (E) Number of symbiont cells per individual planula (n = 5 replicates per depth). (F) Rate of oxygen consumption per individual larva (n = 21 shallow, n = 18 deep). Horizontal black lines within boxes are median values and box limits represent first and third quartiles. Whiskers represent 1.5 times the interquartile range. Round black points are individual sample data. Statistical tests (ANOVA) compare data for shallow versus deep planulae; ns = not significant, ∗ = p < 0.05, ∗∗∗∗ = p < 0.0001.

Newly released coral planulae from deep colonies had significantly smaller volume (0.193 ± 0.009 mm^3^, n = 76) compared to shallow planulae (0.369 ± 0.010 mm^3^, n = 96; One Way ANOVA, F = 60.52, DenDF = 19.44, p < 0.0001) (Figure 1C). Despite this, protein concentration was not significantly different between shallow and deep planulae (Figure 1D). The number of symbiont cells was significantly greater in deep (7140 ± 1846 cells/larva n = 5) compared to shallow planulae (1750 ± 318 cells/larva n = 5; One Way ANOVA, F = 8.27, DenDF = 8, p = 0.021) (Figure 1E). Planulae respiration rate was not significantly different between depths both when normalized to individual specific volume or per individual (Figure 1F).

### Settlement and juvenile physiology

A mixed effects model with well ID (“tank effect”) included as a random factor was selected as the best model for settlement and survival (lowest AIC value, anova {stats}, AIC {stats}). Neither settlement nor survival were significantly influenced by planulae collection date. Using all data i.e., to include all variation in number of larvae per well, probability of settlement and probability of survival were not significantly different between treatments (Kaplan Meier, log rank, p > 0.05) (Figure S2). Differences in the number of larvae in each well plate accounted for 10.4% of the variation in the data (Cox Mixed Effects model fit by maximum likelihood; survival ∼ treatment + (1 | number of larvae per well). Random variance = 0.1039). Kaplan-Meier analysis performed on a subset of the data (n larvae per well = 4–11 inclusive) also returned non-significant p values when comparing treatments. It should be noted that a relatively low number of deep planulae were available for the settlement trials (N = 39, split between two treatments: DD and DS). These numbers may be too small to give robust conclusions and it is desirable to repeat this portion of the experiment with higher replication.

**Figure 2:**
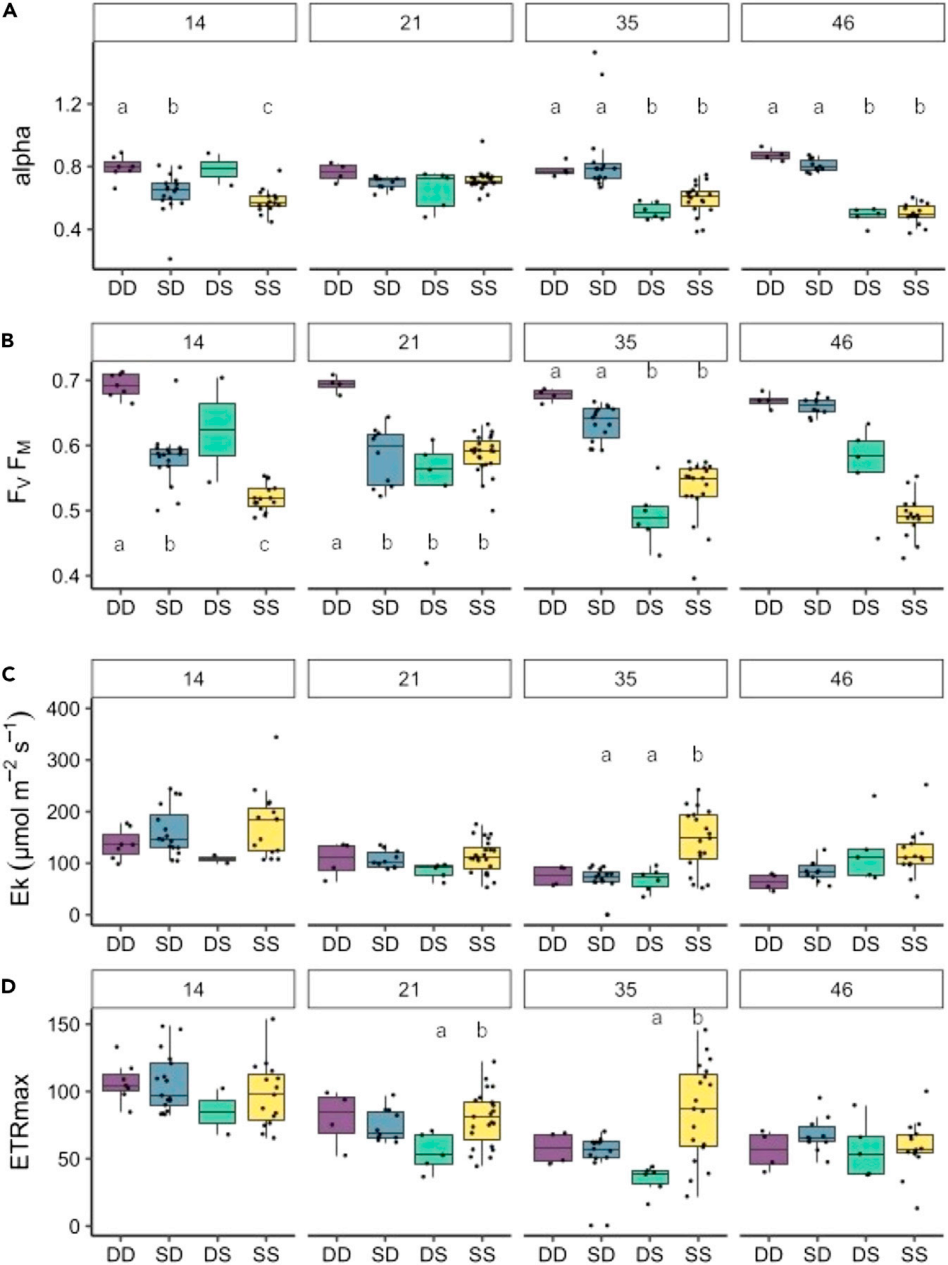
Photochemical parameters of settled spat at five time points where numbers in facet panels refer to days after collection (A–D). Horizontal panels are (A) photosynthetic efficiency at light limiting PAR intensity, (B) maximum quantum yield, (C) saturation irradiance, and (D) maximal relative electron transport rate. Each treatment is represented in all facets on the x axis. Different lower-case letters indicate statistically significant differences between treatments within that sampling time point (adjusted p < 0.05). N for each time/treatment ranges from 2 to 23 spat owing to differential settlement and survival at the time of each measurement. Deep to shallow (DS) spat were not included in statistical tests on day 14 since *n* was less than three. Horizontal black lines within boxes are median values and box limits represent first and third quartiles. Whiskers represent 1.5 times the interquartile range. Round black points are individual sample data.

Alpha is the steepness of the slope between electron transport rate (ETR) values under light limiting conditions; higher values indicate acclimation to low light intensities (Figure 2A). At the earliest time point (14 days after collection), alpha values in each treatment were significantly different from each other, but DD (deep-deep) and DS (deep-shallow) have higher median alpha values than SS (shallow-shallow) and SD (shallow-deep) spat. On day 21, the second time point, there were no statistical differences between the treatments (Kruskal–Wallis test, p > 0.05). By day 35, a different pattern emerged, whereby spat in DD and SD treatments had significantly higher alpha compared to DS and SS (Kruskal–Wallis test, H = 30.03, p < 0.0001). The maximum photosynthetic yield, F_V_/F_M_ (Figure 2B), shows the same pattern as alpha, and from day 35 onwards, the spat exposed to deep light conditions have significantly higher F_V_/F_M_ than those exposed to shallow conditions (DD and SD > SS and DS; Kruskal–Wallis test, H = 34.53, p < 0.0001). This grouping of treatments is sustained and strengthened (lower treatment variability) at the last time point, day 46. F_V_/F_M_ is consistently highest for DD spat (days 14–46 inclusive: 0.68 ± 0.0062). The alpha and F_V_/F_M_ indicate that spat became acclimated to their surrounding environment irrespective of their origin at relatively short temporal scales (5 weeks). Treatment effect on saturating irradiance (E_K_) was less significant though SS spat typically had the highest mean value at each sampling point and significantly higher than DS (Kruskal–Wallis test, H = 16.22, p < 0.05) and SD (Kruskal–Wallis test, H = 14.72, p < 0.01) on day 35. ETR_MAX_ values were more variable showing few significant treatment-induced differences within each day except for DS vs. SS on days 21 and 35.

### Skeletal morphology and growth

**Figure 3:**
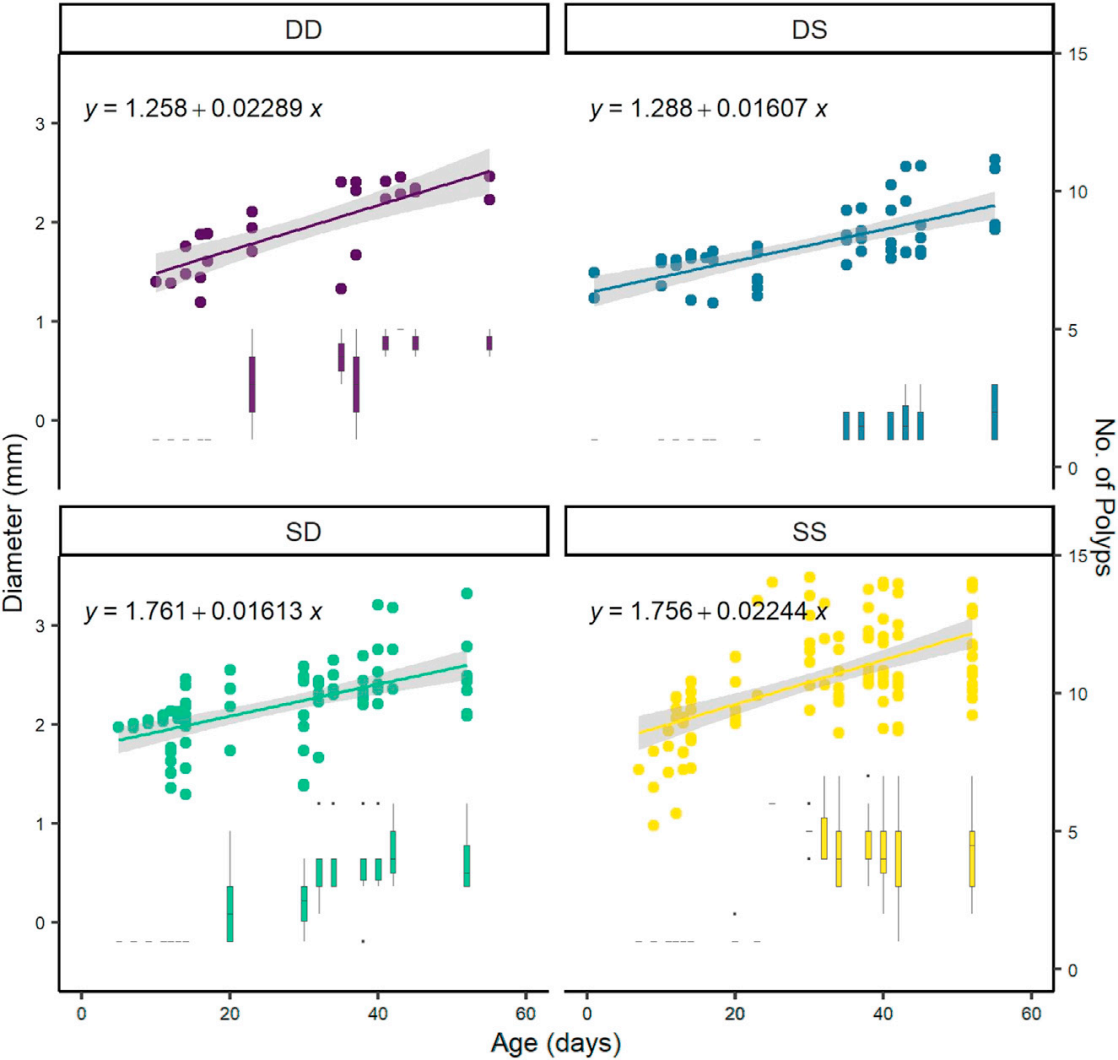
Growth and polyp development of spat in an *ex situ* experiment Points are spat diameter (left axis) with increasing age on the x axis fitted to a linear trend line corresponding to the equation shown in each facet (n individuals per treatment: DD = 4, DS = 6, SD = 10, SS = 14). Gray area around the trend line is 95% confidence interval. Boxplots (right y axis) indicate number of polyps per spat with age, with the central primary polyp after metamorphosis counting as one. Horizontal black lines within boxes are median values and box limits represent first and third quartiles. Whiskers represent 1.5 times the interquartile range.

Planulae originating from the shallow reef had a ca. 28% larger diameter at settlement (lm, y intercept = 1.76 mm both SS and SD) compared to deep origin spat (y intercept = 1.26 mm DD, 1.29 mm DS) (Figure 3). However, corals in both the translocated treatments (i.e., SD and DS) had slower growth rates (reduced linear slope) compared to control corals that remained under light conditions similar to their parental origin (Figure 3). Correspondingly, 55 days after collection, at the termination of the experiment, DS spat (recently settled planulae) had the smallest average diameter (2.27 ± 1.56 mm) and SS spat had the largest average diameter (2.73 ± 1.02 mm, one-way ANOVA, p < 0.05). Spat within the DS treatment were last to asexually produce a single secondary polyp (day 35) and SS spat were the first (day 20). Only in the SS treatment did any of the individual spat produce six complete secondary polyps within the timeframe of the experiment (day 30, 2/29 individuals). Calyx width was significantly larger in SD and SS spat (shallow origin planulae) compared to DD and DS spat (One Way ANOVA, adjusted p value < 0.002). Calyx diameter did not change with spat age i.e., slopes in each treatment were not significantly different from zero (Figure S3).

**Figure 4:**
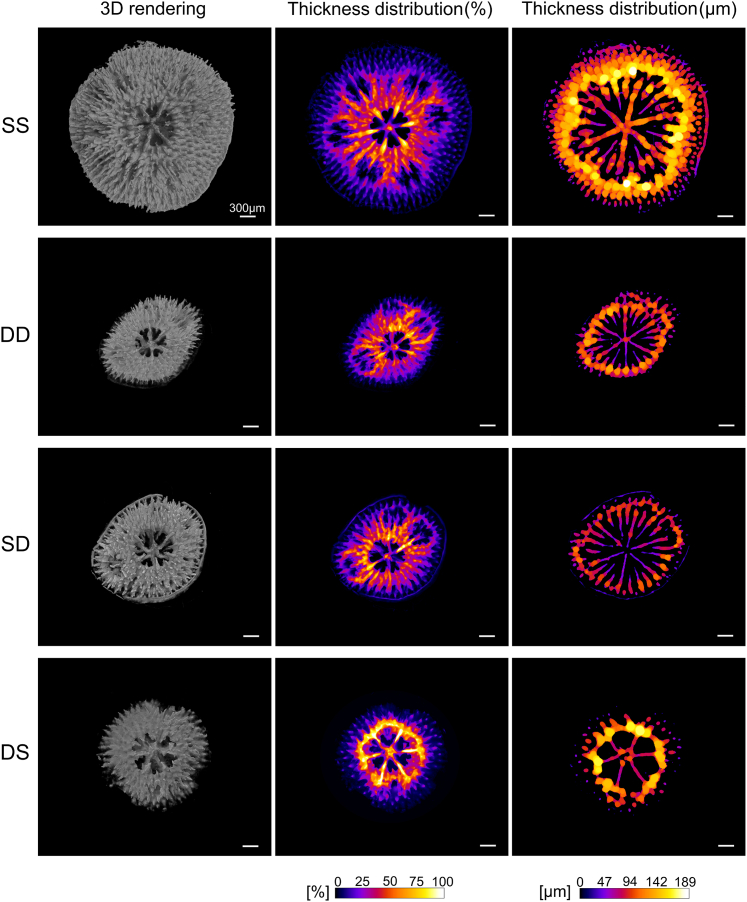
Skeletal morphology of spats imaged using X-ray μCT Left column**:** 3D rendering of the skeleton (top-view); middle column: z-projected thickness distribution (expressed as percentage, each spat’s skeleton, n = 1 per treatment, was normalized to its own 100%); right column: local thickness distribution (expressed as μm, the thickness was projected onto a cross-sectional slice of the skeleton and normalized to the highest value reached across the 4 experimental conditions). Rows show the skeleton of spats from 5 m deep grown under experimental light conditions simulating the shallow (SS) or deep reef (SD) and of spats from 45 m deep grown under experimental light conditions simulating the deep (DD) or shallow reef (DS). All scale bars are 300 μm.

X-ray microCT images of 37-day-old spat (Figure 4) indicate that SS spat had the largest diameter (3.27 mm), greatest number of secondary polyps (n = 6), and greatest skeletal volume (1.52 mm^3^). Skeletal volume was 71% lower for SD (0.44 mm^3^) compared to SS spat. DD and DS spat had 78% (0.34 mm^3^) and 80% (0.31 mm^3^) lower skeletal volume respectively relative to SS spat. Correspondingly, SS spat had the greatest skeletal growth rate (0.0410 mm^3^/day) and DS spat the lowest (0.0083 mm^3^/day). In addition, the spats growing under shallow conditions (SS and DS) had a rounded shape, extending approximately equally in all directions, whereas spats growing in deep conditions (DD and SD) acquired a more oval and elongated shape with a similar arrangement of secondary budding polyps (Figure 4). Shallow growing spat had lower height to width ratios (i.e., more flattened, SS: 0.21, DS: 0.22) than SD (0.32) and DD spat (0.37). Moreover, assessment of local thickness distribution indicates that SS and DS spats possessed a greater thickness of the walls surrounding the calyxes compared to DD and SD spats (Figure 4).

## Discussion

As shallow coral reefs are increasingly degraded by anthropogenic stressors, mesophotic reefs have been highlighted as potential coral refugia.^3^^,^^7^ However, corals and their planulae larvae express significantly different phenotype and morphotype across the depth gradient resulting from contrasting abiotic conditions existing between shallow and deep reefs.^18^^,^^19^^,^^20^^,^^22^^,^^37^^,^^38^ This raises questions about the plasticity of corals to acclimate across this gradient and consequently the possibility for larval supply from deep reefs to reseed degraded shallow reefs. In this study, maternally inherited Symbiodiniaceae endosymbionts of newly settled *S. pistillata* photochemically acclimated to both high and low experimental irradiances within 35 days after settlement (Figures 2A and 2B), despite initially exhibiting photophysiology (Figure 2, day 14) and a larval phenotype significantly dependent on origin depth (Figure 1). Although skeletal morphological acclimation was not evident, irradiance did not reduce the probability of settlement or survival. Such settlement and photophysiological plasticity allowed coral larvae to develop and acclimate to *ex situ* settings where light intensity and spectra differ from their parental origin environment.

### Depth-dependent planulae phenotype

Planulae collected on the shallow reef in this study (5–8 m depth) had a distinct phenotype from mesophotic reef planulae (41–45 m depth). Planulae from the deep reef contained more photosymbionts per individual despite being significantly smaller in volume compared to shallow reef planulae, resulting in a visibly darker color (Figure 1B). Continued tracking of symbiont density in developing spats was not done in the current study, since this is a destructive method, but higher symbiont densities in adult mesophotic corals are a common adaptation to increase light capture at lower light intensities.^18^^,^^39^ Thus, higher symbiont densities in deep origin planulae may prime individuals for life on mesophotic reefs. It was previously reported that deep *S. pistillata* planulae in Eilat host algal symbionts dominantly from the genus *Cladocopium*, whereas shallow planulae host dominantly *Symbiodinium* spp.^25^; the same depth dependent trend is observed in adult colonies of this species at this site.^40^ In addition, planulae with high algal symbiont density have reduced survival at higher temperatures^41^ and since in Eilat the shallow reefs tend to have warmer temperatures than deep reefs,^15^ the retention and settlement of deep origin planulae on deep reefs is further favored. *S. pistillata* has an internal brooding reproductive mode and newly released planulae contain maternally inherited photosymbionts. These specific life history traits likely enhance parental effects and favor larval retention close to the parental colonies^42^^,^^43^^,^^44^ though long distance dispersal of brooded larvae may be possible in some cases.^45^ Indeed population divergence across a depth gradient is more common in brooding species^46^ relative to the approximately 70% of coral species that are broadcast spawners and species for which photosymbionts are acquired 'horizontally' from the environment after settlement.^47^ However, low connectivity and population divergence between shallow and mesophotic corals has been reported for species with a variety of life history traits^46^^,^^48^^,^^49^^,^^50^^,^^51^^,^^52^^,^^53^^,^^54^ meaning that connectivity attained in nature is multifaceted and yet to be fully understood.

Higher symbiont densities and reduced individual size for deep planulae of this species at this site was previously reported^25^ and thus appears to be a conserved trait between cohorts, though mesophotic planulae of other species are not always smaller than shallow conspecifics.^33^ Reduced size is often equated with reduced per offspring investment, and thus the reduced offspring size and adult fecundity at mesophotic depths, as noted for other coral species, is suggested to result from reduced light energy at depth reducing overall energy available for reproduction.^14^^,^^15^^,^^16^^,^^17^^,^^55^ On release, coral planulae are predominantly composed of lipids.^45^^,^^56^^,^^57^^,^^58^ Lipids are the dominant energy source for lecithotrophic coral planulae because translocation from maternally acquired algal symbionts is minimal.^59^ It is likely in the current study that reduced offspring investment to deep planulae was associated with reduced lipid provisions since planulae protein concentration was not significantly depth-dependent (Figure 1D). Though colony size distribution and reef area covered by *S. pistillata* does not decrease and may even increase from shallow to mesophotic depths in the GoA,^5^ reduced fecundity with increasing depth may mean mesophotic *S. pistillata* are at sub-optimal conditions for ecological fitness.

Overall, there is a significant parental effect on planulae phenotype that may predispose planulae for greater fitness and settlement success closer to their parental origin, rather than liberating planulae for wide dispersal into contrasting environments.^30^ Correspondingly, a settlement preference and greater survival rates of juveniles growing within their parental depth range has been noted for corals, with phenotype-environment matching cited as the probable mechanism.^26^ Taken together, these data specifically do not increase the probability for planulae of *S. pistillata* from deep reefs in Eilat to settle on shallow reefs and thus the former acting as a 'refugia' to aid in the recovery of the latter. This point is particularly relevant and timely following the dramatic, near total loss of reef building corals to a depth of ca. 4 m at this study site following a severe storm in March 2020 (*pers. obs*.).

### Retention of parental effect on growth and development rates

Though planulae used for settlement experiments versus volume measurements were obtained from different cohorts (year 2020 versus 2021, respectively), differences in planulae size in 2021 (deep planulae significantly smaller) were evident after metamorphosis in 2020 so that deep individuals had significantly smaller diameters on settlement compared to shallow individuals (Figure 3). Spat translocated away from their parental environment (SD and DS) had the slowest post-settlement growth rates. This means that the translocation itself, rather than light intensity, origin depth, or initial size most strongly dictated lateral growth rates. These data contrast with Shlesinger and Loya^26^ who report that two *Stylophora* species grow fastest on the shallow reef (5 m depth, *in situ*) regardless of whether their origin was shallow or mesophotic, though survival rates were highest for juveniles growing at their parental origin depth. The shallow corals in Shlesinger an Loya’s study were followed over a longer period of three months, likely experienced higher light levels, and perhaps other preferable environmental conditions for growth present *in situ* (e.g., high flow, increased prey availability, macro algae removal by herbivores) but absent in our *ex situ* aquaria. Though the ‘high’ light levels in the current study are likely below those experienced on exposed shallow surfaces *in situ*, the ability to seek shade may enhance the survival of mesophotic planulae on shallow reefs.^34^ The contradicting findings between our studies remain to be resolved. In the current study, microCT imaging also revealed that skeletal volume (a proxy for calcification rate) was lowest in DS spat followed by DD < SD < SS indicating the involvement of both origin and environment. The combination of decreased lateral growth, calcification (skeletal volume), and polyp budding rates (Figure 3) places deep origin planulae that settle at higher light intensities at a size disadvantage compared to shallow reef origin planulae during early ontogeny. Smaller juveniles have higher post-settlement mortality^60^ and colony size determines sexual maturity and onset of egg production in *S. pistillata*.^61^ These results may translate into increased juvenile mortality and delayed sexual maturity for deep and translocated coral colonies, effectively reducing their ecological fitness relative to shallow conspecifics.

Calyx diameter, which in these corals did not significantly change with increasing age in any treatment (Figure S3), was significantly smaller for deep origin corals (origin/parental effect). Smaller calyx diameter is also present in adult colonies of deep relative to shallow colonies of *S. pistillata*^20^; calyx diameters of primary polyps in this study were comparable to those measured for adults of this species.^20^ Smaller calyxes (polyps) may be suggestive of reduced feeding potential, different prey sources, or unrelated to feeding since such a link between calyx size and feeding is seldom reported in corals^62^^,^^63^ but see^64^ for a qualitative observation of differential prey capture between corals with large versus small polyps. Mesophotic coral colonies typically have a higher dependence on heterotrophy rather than translocated photosynthates for energy,^18^^,^^19^^,^^20^^,^^65^^,^^66^ which may be the result of increased feeding effort rather than a morphological adaptation.^62^ Although colony shape and polyp arrangement were most similar between SD and DD spat in mesophotic light conditions (i.e., environmental influence; Figure 4), other growth and skeletal morphology results (spat and calyx diameter) suggest that origin depth exerts a greater influence on *S. pistillata*’s skeleton than ambient light conditions at least in the first two months post-settlement. Reduced growth rates following translocation and limited morphological acclimation to prevailing light conditions within the time frame of our experiment, again favor settlement within the parental environment.

### Symbiodiniaceae versus coral host post-settlement acclimation

Reef building corals host multiple symbiotic organisms, which adds complexity to determining the capacity for corals to adapt to environmental change. How each partner responds to abiotic changes and how those responses influence the interactions between partners dictate corals' ecology.^67^ The most widely researched and energetically important symbionts are the photosynthetic algae from the taxonomic family Symbiodiniaceae. In this study, 14 days after collection, photosynthetic alpha and F_V_/F_M_ values were parental origin dependent. However, from 35 days onwards apparent clustering of photochemical parameters dependent on treatment conditions rather than parental origin depth suggests acclimation of Symbiodiniaceae to respective experimental light conditions (Figures 2A and 2B). This acclimation occurred on a similar timescale to photochemical acclimation seen in an *in situ* depth gradient translocation of adult *S. pistillata* colonies^68^ and appears to persist in longer term field experiments for multiple species.^34^^,^^69^^,^^70^ A decrease in F_V_/F_M_ values of *S. hystrix* juveniles six months after translocation from 40 to 20 m in Okinawa^34^ also agrees with our data showing acclimation potential during early ontogeny. In the current study, an absence of data on potential changes in symbiont density, relative abundance of symbiont species in the coral tissue, or imaging of the photosynthetic apparatus through experimental time, the source mechanism of the observed changes in photochemistry cannot be identified. A previous study at Heron Island, Great Barrier Reef, using three closely related branching corals including *S. pistillata*, reported differing levels of photoacclimation and photoinhibition following depth translocation dependent on whether algal endosymbionts were considered depth generalist or specialist species.^70^ Another study reported a concomitant reduction in symbiont density and maximal photosynthetic efficiency following translocation of adult corals from mesophotic (30 m) to shallow reefs (3 m)^68^ and a positive association exists between these two parameters for multiple species in nature.^71^ Future investigations may choose to revisit the question of how density and species composition of Symbiodiniaceae changes during light acclimation and relate this to any changes in photochemistry.

The acclimation of Symbiodiniaceae to treatment light conditions contrasts the retention of parental origin effects on growth of the host coral skeleton (Figure 3). Symbiodiniaceae may therefore have greater plasticity and faster acclimation capacity compared to the coral host’s ability to modify skeletal features and growth patterns, as previously suggested.^68^ Photoacclimation of Symbiodiniaceae can be observed on a timescale of minutes to hours, for example in response to diel changes in light intensity.^72^^,^^73^^,^^74^^,^^75^ In contrast, whilst diel changes in calcification rate can be observed in corals,^76^ changes in skeletal macro-morphology occur on the timescale of months such as can be seen with seasonal density bands^77^ or changes in corallite shape following translocation to a shallower depth.^78^ In addition, light intensity alone may not drive the morphological differences observed across depth on a natural reef. Specifically, massive and branching growth forms are dominant on shallow reefs versus laminar and encrusting forms beyond 50 m depth.^5^ Species with a wide depth distribution such as *S. pistillata*^36^ and *Montastraea cavernosa*^21^^,^^37^ display morphological variation forming spherical colonies in the shallows and plate-like colonies in the deep. In addition, adult colonies of *S. pistillata* in low light environments have longer, thinner branches and greater branch spacing compared to colonies in high light habitats that results in increased mass transfer and reduced self-shading.^79^^,^^80^ Several studies spanning both branching and massive coral species report smaller calyx diameter, lower calyx height, and greater spacing between calyxes with decreasing light intensity.^20^^,^^21^^,^^78^^,^^81^ These differences in colony-level and calyx-level morphology are commonly reported as light capture adaptations that also reduce self-shading in deep colonies.^82^ However, rounded colonies with thicker branches also better dissipate the higher wave energy on shallow reefs^83^ and taller, larger calyxes increase drag, which may increase prey retention in high flow environments.^84^ Furthermore, colonies with larger calyxes are better at sediment removal, which may be a more pervasive issue in high energy, shallow reefs.^85^ Though light intensity is typically considered the dominant driver of calyx-level changes,^78^^,^^82^ it is possible that the absence of differential flow or sedimentation or the relatively short experimental period (2 months) precluded the observation of morphological acclimation in this *ex situ* experiment and is a consideration for future experiments.

### Potential for population connectivity across depth

Of all treatments, DS spat were smallest in diameter at the end of the experiment (55 days), with slowest growth and polyp budding rates, reduced skeletal volume, and small disordered skeletal features (Figures 3 and 4). These observations do not lend support to the hypothesis that coral planulae originating from deep reefs have the morphological plasticity to replace the ecosystem function and niche of shallow corals.^6^^,^^7^ Furthermore, other recent studies report significantly decreased settlement and survival rates of mesophotic *S. hystrix*^34^ and *S. kuehlmanni*^26^ juveniles when exposed to high light/shallow reef conditions. Although, in this study, planulae under conditions different from their parental origin (both DS and SD) did not suffer reduced settlement success or survival in contrast to the aforementioned studies and showed photochemical acclimation, growth rates in these treatments were significantly reduced compared to corals retained in experimental conditions more like their parental origin. In addition, *in situ* coral planulae are faced with intra- and inter-specific competition. Conducting the larval settlement phase of translocation experiments *in situ*, should be a goal, albeit a logistically challenging one, for future studies. Reduced growth rates are expected to decrease survival *in situ*, for example, by limiting competitive advantage to occupy space, and reductions in skeletal volume and thickness may confer increased sensitivity to mechanical stressors or grazing. Overall, a lack of potential for mesophotic larvae to settle and persist on shallow reefs is a conclusion shared by multiple previous studies,^46^^,^^48^^,^^49^^,^^50^^,^^51^^,^^52^^,^^53^^,^^54^ though Sturm et al.^86^ present an interesting consideration for connectivity between mesophotic and down current shallow populations for the broadcast spawner *M. cavernosa* in the Florida Keys. Future studies should attempt to evaluate population connectivity for multiple species in Eilat, with a range of life history traits, either through modeling or genetic methods. Such studies may elucidate coral trait or environmental limitations for connectivity across depth and may define the depth of optimal fitness for investigated species, which is of particular interest in species presently considered as “depth generalists”.

Larger size at settlement and rapid symbiont photochemical acclimation of shallow origin planulae, together with the known dominant downwelling water convection on the western side of the Gulf of Aqaba^87^ amplifies the probability that, rather than being a source, mesophotic reefs of Eilat may currently act as a larval sink for multiple coral taxa including *Stylophora*.^26^^,^^88^ This dominant one-way flux of coral planulae from shallow to deep reefs resembles previous studies including other regions and coral species^26^^,^^46^^,^^48^^,^^54^ and does not lend support to Deep Reef Refugia Hypothesis. However, although DD spat were small at time of settlement, high F_V_/F_M_, alpha, survival, and growth rates confer relatively good growth potential for DD spat on deep, low light reefs. Furthermore, mesophotic reefs of Eilat are comparatively sheltered from shallow reef disturbances with lower thermal variability,^15^ which, locally, may permit higher coral survival^88^ and abundance on deep reefs.^5^ This potential, in addition to other unique qualities of mesophotic coral ecosystems, makes them worthy of protection irrespective of their connectivity to shallow reefs. Recently, the Mediterranean coral *Oculina patagonica* was observed at mesophotic depths having previously been exclusively a shallow water species in Israeli waters.^89^ The authors suggest its migration is a response to rapidly increasing sea surface temperatures in the Mediterranean and, though shallow reef colonies of *S. pistillata* in Eilat appear resistant to 4–5°C above summer maximum temperatures,^72^ shallow reefs face additional threats such as storm damage. Taken together, mesophotic reefs may become an increasingly dominant sink for coral planulae and play a role in preventing the local extinction of the study species. However, the proposition of the Deep Reef Refuge Hypothesis that mesophotic coral larvae can replenish shallow reefs is not supported by the current study.

### Limitations of the study

Owing to reduced fecundity of *S. pistillata* with increasing depth, fewer deep origin planulae were used in this study relative to shallow planulae, despite increased sampling effort at 45 m relative to 5 m depth. The discrepancy in sample size is particularly notable in settlement assays. The statistical result of no significant differences in settlement and mortality rates between treatments should therefore be interpreted with caution. Future studies should aim to readdress this question to assess the biological significance of planulae translocation on survival rates.

Second, because this was an *ex situ* aquaria study, not all abiotic and biotic characteristics of a natural reef were present. For example, wave action, variable water flow, sedimentation, and competition influence morphological development in nature. Observations that differ from the present study may be obtained *in situ*. Finally, the experimental light intensity used for the high light treatment was likely lower than at the parental origin/planulae collection depth in Eilat. Though light intensity was consistent across high light treatments and throughout the experiment, this potentially altered acclimation pressures that planulae were exposed to. Future experiments should continuously seek to mimic *in situ* conditions as far as possible.

## STAR★Methods

### Key resources table

**Table:** 

REAGENT or RESOURCE	SOURCE	IDENTIFIER
**Biological samples**		

*Stylophora pistillata*larvae	Eilat reef (5 and 45 m depth), Gulf of Aqaba/Eilat, Northern Red Sea (29°30′05.9"N 34°55′01.7"E)	Israeli Nature Parks Authority Permit number 2020/42677.

**Chemicals, peptides, and recombinant proteins**		

Bradford Protein Assay	Bio-Rad	5000001

**Deposited data**		

Original data and code	Github, Zenodo	Github: https://github.com/JessicaBellworthy/Bellworthy_planulae_exsitu_translocation; Zenodo: https://doi.org/10.5281/zenodo.7763243

**Software and algorithms**		

ImageJ	ImageJ GitHub	version 1.53
R (A language and environment for statistical computing)	R Core Team	version 4.0.3
FIJI	ImageJ GitHub	version 1.53

**Other**		

GHL Mitras LX 7206 LED aquarium light system	Bulk Reef Supply	SKU 250119
80μL-well microplate sensor dish reader	Loligo Systems	CH25000
Binocular microscope	Nikon	Model: MultiZoom Az-100
Pulse Amplitude Modulated Fluorometer	Walz-GMbH	Imaging PAM
Imaging beamline	Helmholtz-Zentrum Berlin, Germany	BAMline at BESSY

### Resource availability

#### Lead contact

Further information and requests for resources should be directed to and will be fulfilled by the corresponding author, Jessica Bellworthy (jhbellworthy@gmail.com).

#### Materials availability

This study did not generate any new materials or reagents.

### Experimental model

This study collected planulae larvae from visibly healthy adult colonies of the hermaphrodite scleractinian coral *Stylophora pistillata* (Esper, 1792).

### Method details

#### Larval collection

Coral planulae (larvae) were collected from the reef adjacent to the Interuniversity Institute in Eilat, Israel, under a permit from the Israeli Natural Parks Authority. Planulae were collected from 5-8 meters (shallow) and 41-45 meters (deep) water depths, using recreational open circuit SCUBA and Closed-Circuit Rebreather diving, respectively. Traps made of plankton net (200μm mesh) and a funnel attached to a collection bottle were placed over *Stylophora pistillata* colonies at each depth an hour before sunset and removed the following morning shortly after sunrise. The use of these traps over individual colonies at each depth ensures the origin of the planulae i.e., only planulae from each colony will be retained within the trap. Planulae were collected on 11^th^ May and 10^th^ June 2020 and used for an *ex situ* settlement experiment. Planulae were distributed to every treatment on each sampling date; the second sampling served to increase the number of replicates. Additional planulae were collected from both depths in the same manner over five nights between 26^th^ April and 24^th^ May 2021 for the assessment of larval physiology and respiration rate. In total, 69 traps were set on the shallow reef; approximately 40% of the traps contained planulae (range 1 - >200 per net, N total ca. 1600 planulae). A total of 115 traps were set on the deep reef; approximately 30% of the traps contained planulae (range 1 – ca. 50 per net, N total ca. 750 planulae). Planulae from these collections were distributed between four graduate and intern experiments, so exact numbers of planulae used for each analysis are given in the methods sections below. Planulae from different colonies and different sampling days were pooled by collection depth for all analyses and sampling day was incorporated as a random factor during statistical analyses (see below).

#### Experimental set up

Live coral planulae were transported to the University of Haifa, northern Israel on the day of collection in ambient filtered seawater (0.45μm) from the collection site (Eilat). Time from collection to arrival in at the University of Haifa was less than 12 hours and mortality and settlement on route was ‘minimal’ (*author observation*). Planulae pooled by treatment were placed in 6-well plates with the lids attached with Parafilm (Sigma-Aldrich) and submerged in flow-through aquaria mimicking northern Red Sea conditions at the time of collection (pH 8.2, salinity 40 g L^-1^, temperature 24°C). Well plates were immersed in treatment aquaria to accumulate biofilm for one week prior to introducing the planulae. Water flow was created by incoming circulating artificial seawater (Red Sea Salt, Red Sea Ltd) and a wave maker pump (EcoDrift 4.0, AquaMedic). Planulae from each collection depth were placed under light intensity and spectra conditions referred to as ‘shallow’ (high light intensity, wide light spectrum) or ‘deep’ (low light intensity, narrow light spectrum) in a reciprocal manner. We chose to focus upon light manipulation since this abiotic factor has a steep gradient between shallow and mesophotic reefs in Eilat decreasing from ca. 55% of surface values at 5 meters (photosynthetically active radiation) to ca. 4% at 40 meters depth^91^^,^^92^ and light has a vital role in the ecology of symbiotic corals. Specifically, half of the planulae from the shallow collection depth remained in ‘shallow’ light conditions (250 μmol m^-2^ s^-1^, shallow-shallow: SS; N = 146 planulae, 10 wells, 6 – 25 planulae/ well) whilst the other half were placed in ‘deep’ light conditions (shallow-deep: SD; N = 146 planulae, 10 wells, 6 – 25 planulae/ well). Similarly, half of the planulae collected from below 40 meters remained in ‘deep’ light conditions (20 μmol m^-2^ s^-1^) as a deep control (deep-deep: DD; N = 19 planulae, 5 wells, 1 – 6 planulae/ well) or were placed under ‘shallow’ light conditions (deep-shallow: DS; N = 20 planulae, 5 wells, 1 – 7 planulae/ well). Once all planulae had settled, lids were removed from the settlement plates. A 12-h/12-h photoperiod with light spectra corresponding to each origin depth^92^ was provided by a Mitras LX 7206 LED aquarium light system (GHL). Spectral irradiance in ‘shallow’ aquaria ranged between ∼100 μW cm^−2 nm−1^ at 400 nm and ∼40 μW cm^−2 nm−1^ at 650 nm. In ‘deep’ aquaria spectral irradiance ranged between ∼5 μW cm^−2 nm−1^ at 400nm and ∼1 μW cm^−2 nm−1^ at 650 nm.^92^ The spectral distribution of the lights is presented in Figure S1. ‘Shallow’ aquaria therefore had a light intensity 12.5 times higher than ‘deep’ aquaria and spectra closely reflected the *in situ* conditions at the collection depth.^92^ Whilst the light intensity used in ‘shallow’ aquaria may be considered relatively low for 5 m depth at the planulae collection site (Eilat, Gulf of Aqaba), the intensity (together with the well-matched spectrum) may be considered as a simulation of a partially shaded ‘shallow’ reef which coral planulae under high light conditions typically seek.^93^^,^^94^^,^^95^^,^^96^^,^^97^ Variable sample sizes were an unavoidable result of differences in the total number of larvae collected from each depth, resulting in an unbalanced design. Triplicate aquaria for were used for each light condition with planulae distributed as evenly as possible between aquaria.

#### Larval physiology

Immediately post collection in 2021, planulae pooled by collection depth were photographed under a light microscope (n shallow = 96, n deep = 76). Images were scaled with ImageJ and the length and width of planulae were measured. Larval volume was calculated as for an elliptical sphere V = 4/3 π*ab*^2^ where *a* is half the planulae width and *b* is half the length.^28^ Only elongated, cylindrical-shaped planulae were measured excluding planulae that had begun metamorphosis; our analysis therefore excluded visibly damaged or spherical shaped individuals. Excess seawater was then removed from imaged planulae and batches of 20 individuals were frozen at −80°C for downstream measurement of total protein concentration using the Bradford protocol^98^ and symbiont density counted with a haemocytometer (n replicate batches of 20 planulae shallow = 5, deep = 5). Details of protein concentration and cell count protocols have been previously described.^99^ Additional planulae were placed into 80μL-well microplate sensor dish reader (SDR, Loligo Systems), one individual planula per well, to measure holobiont respiration. Respiration rate for each well (planula) was determined using a least squares linear regression of oxygen concentration per unit time in R v.4.0.3^100^ with the package ‘LoLinR’ (v0.0.0.9000,^101^). Samples with non-negative rates were excluded (n excluded shallow = 2, deep = 2) resulting in n = 21 shallow planulae and n = 18 deep planulae. Mean blank rate (n blanks = 16) was subtracted from each sample's respiration rate, which was expressed as nmol O_2_ larva^-1^ min^-1^. All physiological data were normalised to number of planulae in the sample e.g., protein concentration per individual larva*.*

#### Juvenile physiology

Sampling time points are referred to as 'days post collection' with day zero being the day of collection. Settlement and mortality rates of all planulae in the aquaria experiment were checked at 1 – 3-day intervals from zero to 24 days post collection. Settlement was defined as the completion of metamorphosis and attachment to the substrate. Following settlement, lids were removed and well plates were submerged in aquaria. Survival counts included remaining swimming planulae and settled spat with visible live tissue. In addition, photographs were taken using a binocular microscope (MultiZoom Az-100, Nikon) to track specific settled individuals (spat) at 2 – 5-day intervals over 55 days post collection to monitor radial growth rate, the development of new polyps, and calyx diameter (n individuals per treatment: DD = 4, DS = 6, SD = 10, SS = 14). Spat were measured from scaled photographs at their widest diameter using ImageJ. Growth rates were determined from a linear trend line fitted to the data. The development of new polyps was recorded once six septa could be observed within the new calyx on binocular images. Calyx diameter (inner diameter, wall to wall at the widest point) was determined on the central primary polyp calyx only.

At 14-, 21-, 35-, and 46-days post collection the photochemical efficiency of settled spat was assessed using Imaging PAM fluorometry (Walz-GMbH). Spat were first dark acclimated for 20 minutes before quantum yield was determined with a saturating light pulse following 3 minutes of actinic light illumination in 13 steps of increasing intensity (0, 1, 11, 21, 55, 81, 111, 146, 231, 336, 461, 611, 926 μmol m^-2^ s^-1^. Instrument settings: measuring light = 2, frequency = 1, gain = 3, damping = 2, actinic light = 8, width = 0, saturation pulse intensity = 10). Relative electron transport rate (*r*ETR) was calculated as Y(II)∗PAR for each light step. R Studio was used to fit curves to rapid light curve data^102^ and extract the parameters alpha (photosynthetic efficiency under light limiting irradiances), rETRmax (maximum relative electron transport rate), and Ek (saturation irradiance) using the fitPGH function from the phytotools package as previously adapted and described in detail.^103^^,^^104^ One outlier value (>700 μmol m^-2^ s^-1^; SD, day 14) was removed from the Ek data. Y(II) following a saturation pulse where PAR = 0 is described as maximum quantum yield (F_V_/F_M_).

#### Synchrotron-based X-Ray microCT

High-resolution X-ray microCT of 37 day old spat was conducted at BAMline,^105^^,^^106^ the imaging beamline at BESSY (HZB - Helmholtz-Zentrum Berlin, Germany). One random spat from each treatment was removed from storage in ethanol, attached to a metal stub, and scanned with repeated rotation (spanning 180°) on a high-resolution imaging sample stage.^107^ Only one spat from each treatment was imaged due to limited available time on the synchrotron and the multiple hours required for imaging each sample. Energy in the range of 15keV to 25keV were applied, with the exposure times set to 1 sec. Projection images were acquired with a final pixel size of 2.2 μm. Data pre-processing and reconstruction were performed using an in-house Octave-based reconstruction pipeline in the labs of the Charité, Universitätsmedizin Berlin. Specifically, the radiograms were background-corrected by normalization with empty beam (flat-field) images, obtained intermittently, after subtraction of dark-current images. Filtered back projection was computed using nRecon (v 1.7.4.2, Brucker micro-CT, Belgium). Data were viewed in 3D using CTvox (v 3.0, Brucker-microCT, Belgium) and analysed as a stack in FIJI^108^ to visualize the z-projected thickness distribution of the skeleton (expressed as percentage, each spat’s skeleton was normalized to its own 100%). In FIJI, thresholding on the z-stack was performed using the Otsu approach^109^ and the voxel counter plug-in was used to determine the total volume of the skeleton (only mineral, without cavities) for each spat. In addition, the local thickness plug-in was applied to the thresholded z-stack to compute the skeletal thickness in micrometres (μm), which was then normalized to the highest thickness value reached among all the imaged spats.

### Quantification and statistical analyses

The number of replicates for each analysis was maximised based on the availability of planulae/ spat in each treatment often resulting in an unbalanced design. All data exploration, graphics, and statistical analyses were performed using RStudio (v4.0.3). Functions and packages used are denoted as ('function' {'package'}) in text. Parametric assumptions of homogenous variance between groups and normally distributed data within groups were tested using Levene's Test and a Shapiro-Wilk Normality Test, respectively.

Since planulae for estimation of larval volume and respiration were collected over multiple days and planulae physiology has been shown to vary between release days,^110^ the day of collection was initially incorporated into Linear Mixed Effect models as a random factor (lmer {lme4}).^111^ In both cases, the Mixed Effect model did not fit the data significantly better than a simple linear model (lm, anova {stats}).^100^ The simplified reduced linear models (One Way ANOVA) were therefore used to infer statistical differences between the physiology of deep and shallow collected planulae. Linear models (size∼age) with Dunn’s Kruskal-Wallis multiple comparisons post hoc test (dunnTest {FSA})^112^ were used to test significant differences between treatments in calyx width and spat diameter. One way ANOVA with Tukey's multiple comparisons tests were used on photochemical data except when data groups did not conform to parametric assumptions; in that case Kruskal-Wallis and Dunn's multiple comparisons tests were used. Differences between treatments were considered significant when the Holm's adjusted p value was <0.05.

Kaplan-Meier survival curves were created (surv_fit {survminer})^113^ and visualised (ggsurvplot {survminer})^113^ to assess differences in time to settlement and mortality rates between the four treatments (log rank test). Due to the range in the number of larvae initially placed in each well and possible density dependant survival of settlers,^114^^,^^115^ data were extensively explored to assess the contribution of random effects including number of larvae per well, individual well plate, aquaria, and start date to model variance (coxme {coxme}).^116^ For this data exploration, mixed effect models were compared based on AIC values (AIC {stats})^100^ and the simplest model chosen in all cases.

All graphics were produced ggplot2^117^ and arranged cowplot^118^ with R Studio. Final aesthetic modifications on graphics were made with the freeware GIMP (v2.10.28). Data are mean ± standard error unless otherwise written. Differences are described as significant when p < 0.05.

## Data Availability

All data and R code to replicate the analyses and construct the raw graphics are publicly available on GitHub (https://github.com/JessicaBellworthy/Bellworthy_planulae_exsitu_translocation).^90^ Raw data and code are also available from Zenodo: 10.5281/zenodo.7763242. Any additional information required to reanalyse the data reported in this paper is available from the lead contact upon request.

## References

[1] Hughes T.P., Barnes M.L., Bellwood D.R., Cinner J.E., Cumming G.S., Jackson J.B.C., Kleypas J., Van De Leemput I.A., Lough J.M., Morrison T.H. (2017). Coral reefs in the anthropocene. Nature.

[2] Carpenter K.E., Abrar M., Aeby G., Aronson R.B., Banks S., Bruckner A., Chiriboga A., Cortés J., Delbeek J.C., DeVantier L. (2008). One-third of reef-building corals face elevated extinction risk from climate change and local impacts. Science.

[3] Hoegh-Guldberg O., Poloczanska E.S., Skirving W., Dove S. (2017). Coral reef ecosystems under climate change and ocean acidification. Front. Mar. Sci..

[4] Bak R.P.M., Nieuwland G., Meesters E.H. (2005). Coral reef crisis in deep and shallow reefs: 30 years of constancy and change in reefs of Curacao and Bonaire. Coral Reefs.

[5] Kramer N., Tamir R., Eyal G., Loya Y. (2020). Coral morphology portrays the spatial distribution and population size-structure along a 5–100 m depth gradient. Front. Mar. Sci..

[6] Glynn P.W. (1996). Coral reef bleaching: facts, hypotheses and implications. Global Change Biol..

[7] Bongaerts P., Ridgway T., Sampayo E.M., Hoegh-Guldberg O. (2010). Assessing the ‘deep reef refugia’ hypothesis: focus on Caribbean reefs. Coral Reefs.

[8] Rocha L.A., Pinheiro H.T., Shepherd B., Papastamatiou Y.P., Luiz O.J., Pyle R.L., Bongaerts P. (2018). Mesophotic coral ecosystems are threatened and ecologically distinct from shallow water reefs. Science.

[9] Smith T.B., Gyory J., Brandt M.E., Miller W.J., Jossart J., Nemeth R.S. (2016). Caribbean mesophotic coral ecosystems are unlikely climate change refugia. Global Change Biol..

[10] Nir O., Gruber D.F., Shemesh E., Glasser E., Tchernov D. (2014). Seasonal mesophotic coral bleaching of Stylophora pistillata in the northern Red Sea. PLoS One.

[11] Kubomura T., Yamashiro H., Reimer J.D. (2018). Appearance of an anomalous black band disease at upper mesophotic depths after coral bleaching. Dis. Aquat. Org..

[12] Williams S.M., García-Sais J., Sabater-Clavell J. (2021). Prevalence of stony coral tissue loss disease at el seco, a mesophotic reef system off vieques Island, Puerto Rico. Front. Mar. Sci..

[13] Morais J., Santos B.A. (2022). Prevalence and extent of coral diseases in shallow and mesophotic reefs of the Southwestern Atlantic. Coral Reefs.

[14] Liberman R., Shlesinger T., Loya Y., Benayahu Y. (2018). Octocoral sexual reproduction: temporal disparity between mesophotic and shallow-reef populations. Front. Mar. Sci..

[15] Shlesinger T., Grinblat M., Rapuano H., Amit T., Loya Y. (2018). Can mesophotic reefs replenish shallow reefs? Reduced coral reproductive performance casts a doubt. Ecology.

[16] Feldman B., Shlesinger T., Loya Y. (2018). Mesophotic coral-reef environments depress the reproduction of the coral Paramontastraea peresi in the Red Sea. Coral Reefs.

[17] Prasetia R., Sinniger F., Hashizume K., Harii S. (2017). Reproductive biology of the deep brooding coral Seriatopora hystrix: implications for shallow reef recovery. PLoS One.

[18] Mass T., Einbinder S., Brokovich E., Shashar N., Vago R., Erez J., Dubinsky Z. (2007). Photoacclimation of *Stylophora pistillata* to light extremes: metabolism and calcification. Mar. Ecol. Prog. Ser..

[19] Martinez S., Kolodny Y., Shemesh E., Scucchia F., Nevo R., Levin-Zaidman S., Paltiel Y., Keren N., Tchernov D., Mass T. (2020). Energy sources of the depth-generalist mixotrophic coral *Stylophora pistillata*. Front. Mar. Sci..

[20] Einbinder S., Mass T., Brokovich E., Dubinsky Z., Erez J., Tchernov D. (2009). Changes in morphology and diet of the coral *Stylophora pistillata* along a depth gradient. Mar. Ecol. Prog. Ser..

[21] Goodbody-Gringley G., Waletich J. (2018). Morphological plasticity of the depth generalist coral, *Montastraea cavernosa*, on mesophotic reefs in Bermuda. Ecology.

[22] Malik A., Einbinder S., Martinez S., Tchernov D., Haviv S., Almuly R., Zaslansky P., Polishchuk I., Pokroy B., Stolarski J., Mass T. (2021). Molecular and skeletal fingerprints of scleractinian coral biomineralization: from the sea surface to mesophotic depths. Acta Biomater..

[23] Scucchia F., Malik A., Putnam H.M., Mass T. (2021). Genetic and physiological traits conferring tolerance to ocean acidification in mesophotic corals. Global Change Biol..

[24] Roberts T.E., Bridge T.C.L., Caley M.J., Madin J.S., Baird A.H. (2019). Resolving the depth zonation paradox in reef-building corals. Ecology.

[25] Scucchia F., Nativ H., Neder M., Goodbody-Gringley G., Mass T. (2020). Physiological characteristics of *Stylophora pistillata* larvae across a depth gradient. Front. Mar. Sci..

[26] Shlesinger T., Loya Y. (2021). Depth-dependent parental effects create invisible barriers to coral dispersal. Commun. Biol..

[27] Graham E.M., Baird A.H., Connolly S.R., Sewell M.A., Willis B.L. (2017). Uncoupling temperature-dependent mortality from lipid depletion for scleractinian coral larvae. Coral Reefs.

[28] Isomura N., Nishihira M. (2001). Size variation of planulae and its effect on the lifetime of planulae in three pocilloporid corals. Coral Reefs.

[29] Hata T., Madin J.S., Cumbo V.R., Denny M., Figueiredo J., Harii S., Thomas C.J., Baird A.H. (2017). Coral larvae are poor swimmers and require fine-scale reef structure to settle. Sci. Rep..

[30] Crean A.J., Marshall D.J. (2009). Coping with environmental uncertainty: dynamic bet hedging as a maternal effect. Philos. Trans. R. Soc. Lond. B Biol. Sci..

[31] Uller T. (2008). Developmental plasticity and the evolution of parental effects. Trends Ecol. Evol..

[32] Wong K.H., Goodbody-Gringley G., de Putron S.J., Becker D.M., Chequer A., Putnam H.M. (2021). Brooded coral offspring physiology depends on the combined effects of parental press and pulse thermal history. Global Change Biol..

[33] Goodbody-Gringley G., Scucchia F., Ju R., Chequer A., Einbinder S., Martinez S., Nativ H., Mass T. (2021). Plasticity of *Porites astreoides* early life history stages suggests mesophotic coral ecosystems act as refugia in Bermuda. Front. Mar. Sci..

[34] Prasetia R., Sinniger F., Nakamura T., Harii S. (2022). Limited acclimation of early life stages of the coral Seriatopora hystrix from mesophotic depth to shallow reefs. Sci. Rep..

[35] Loya Y. (1976). The Red Sea coral *Stylophora pistillata* is an r strategist. Nature.

[36] Einbinder S., Gruber D.F., Salomon E., Liran O., Keren N., Tchernov D. (2016). Novel adaptive photosynthetic characteristics of mesophotic symbiotic microalgae within the reef-building coral, Stylophora pistillata. Front. Mar. Sci..

[37] Goodbody-Gringley G., Marchini C., Chequer A.D., Goffredo S. (2015). Population structure of *Montastraea cavernosa* on shallow versus mesophotic reefs in Bermuda. PLoS One.

[38] Goodbody-Gringley G., Wong K.H., Becker D.M., Glennon K., de Putron S.J. (2018). Reproductive ecology and early life history traits of the brooding coral, *Porites astreoides*, from shallow to mesophotic zones. Coral Reefs.

[39] Titlyanov E.A., Titlyanova T.V., Yamazato K., Van Woesik R. (2001). Photo-acclimation of the hermatypic coral Stylophora pistillata while subjected to either starvation or food provisioning. J. Exp. Mar. Biol. Ecol..

[40] Byler K.A., Carmi-Veal M., Fine M., Goulet T.L. (2013). Multiple symbiont acquisition strategies as an adaptive mechanism in the coral *Stylophora pistillata*. PLoS One.

[41] Yakovleva I.M., Baird A.H., Yamamoto H.H., Bhagooli R., Nonaka M., Hidaka M. (2009). Algal symbionts increase oxidative damage and death in coral larvae at high temperatures. Mar. Ecol. Prog. Ser..

[42] Padilla-Gamiño J.L., Pochon X., Bird C., Concepcion G.T., Gates R.D. (2012). From parent to gamete: vertical transmission of symbiodinium (dinophyceae) ITS2 sequence assemblages in the reef building coral montipora capitata. PLoS One.

[43] Ritson-Williams, R., Arnold, S.N., Fogarty, N.D., Steneck, R.S., Vermeij, M.J.A., and Paul, V.J. (2009). New Perspectives on Ecological Mechanisms Affecting Coral Recruitment on Reefs NSUWorks Citation.

[44] Carlon D.B., Olson R.R. (1993). Larval dispersal distance as an explanation for adult spatial pattern in two Caribbean reef corals. J. Exp. Mar. Biol. Ecol..

[45] Richmond R.H. (1987). Energetics, competency, and long-distance dispersal of planula larvae of the coral Pocillopora damicornis. Mar. Biol..

[46] Bongaerts P., Riginos C., Brunner R., Englebert N., Smith S.R., Hoegh-Guldberg O. (2017). Deep reefs are not universal refuges: reseeding potential varies among coral species. Sci. Adv..

[47] Baird A.H., Guest J.R., Willis B.L. (2009). Systematic and biogeographical patterns in the reproductive biology of scleractinian corals. Annu. Rev. Ecol. Evol. Syst..

[48] Prada C., Hellberg M.E. (2013). Long prereproductive selection and divergence by depth in a Caribbean candelabrum coral. Proc. Natl. Acad. Sci. USA.

[49] Eytan R.I., Hayes M., Arbour-Reily P., Miller M., Hellberg M.E. (2009). Nuclear sequences reveal mid-range isolation of an imperilled deep-water coral population. Mol. Ecol..

[50] Brazeau D.A., Lesser M.P., Slattery M. (2013). Genetic structure in the coral, Montastraea cavernosa: assessing genetic differentiation among and within mesophotic reefs. PLoS One.

[51] Van Oppen M.J.H., Bongaerts P., Underwood J.N., Peplow L.M., Cooper T.F. (2011). The role of deep reefs in shallow reef recovery: an assessment of vertical connectivity in a brooding coral from west and east Australia. Mol. Ecol..

[52] Eckert R.J., Studivan M.S., Voss J.D. (2019). Populations of the coral species Montastraea cavernosa on the Belize Barrier Reef lack vertical connectivity. Sci. Rep..

[53] Serrano X.M., Baums I.B., Smith T.B., Jones R.J., Shearer T.L., Baker A.C. (2016). Long distance dispersal and vertical gene flow in the Caribbean brooding coral Porites astreoides. Sci. Rep..

[54] Serrano X., Baums I.B., O’Reilly K., Smith T.B., Jones R.J., Shearer T.L., Nunes F.L.D., Baker A.C. (2014). Geographic differences in vertical connectivity in the Caribbean coral Montastraea cavernosa despite high levels of horizontal connectivity at shallow depths. Mol. Ecol..

[55] Bloomberg J., Holstein D.M. (2021). Mesophotic coral refuges following multiple disturbances. Coral Reefs.

[56] Arai I., Kato M., Heyward A., Ikeda Y., Iizuka T., Maruyama T. (1993). Lipid composition of positively buoyant eggs of reef building corals. Coral Reefs.

[57] Rivest E.B., Hofmann G.E. (2015). Effects of temperature and *p*CO2 on lipid use and biological parameters of planulae of *Pocillopora damicornis*. J. Exp. Mar. Biol. Ecol..

[58] Ben-David-Zaslow R., Benayahu Y. (2000). Biochemical composition, metabolism, and amino acid transport in planula-larvae of the soft coral *Heteroxenia fuscescens*. J. Exp. Zool..

[59] Kopp C., Domart-Coulon I., Barthelemy D., Meibom A. (2016). Nutritional input from dinoflagellate symbionts in reef-building corals is minimal during planula larval life stage. Sci. Adv..

[60] Raymundo L.J., Maypa A.P. (2004). Getting bigger faster: mediation of size-specific mortality via fusion in juvenile coral transplants. Ecol. Appl..

[61] Rinkevich B., Loya Y. (1979). The reproduction of the Red Sea coral Stylophora pistillata. II. Synchronization in breeding and seasonality of planulae shedding. Mar. Ecol. Prog. Ser..

[62] Palardy J.E., Grottoli A.G., Matthews K.A. (2005). Effects of upwelling, depth, morphology and polyp size on feeding in three species of Panamanian corals. Mar. Ecol. Prog. Ser..

[63] Grottoli A.G., Wellington G.M. (1999). Effect of light and zooplankton on skeletal δ13C values in the eastern Pacific corals *Pavona clavus* and *Pavona gigantea*. Coral Reefs.

[64] Lindemann Y., Eyal G., Genin A. (2019). Intense capture of swarming pteropods by large-polyp corals. Galaxea, J. Coral Reef Stud..

[65] Lesser M.P., Slattery M., Stat M., Ojimi M., Gates R.D., Grottoli A. (2010). Photoacclimatization by the coral Montastraea cavernosa in the mesophotic zone: light, food, and genetics. Ecology.

[66] Padilla-Gamiño J.L., Roth M.S., Rodrigues L.J., Bradley C.J., Bidigare R.R., Gates R.D., Smith C.M., Spalding H.L. (2019). Ecophysiology of mesophotic reef-building corals in Hawai‘i is influenced by symbiont–host associations, photoacclimatization, trophic plasticity, and adaptation. Limnol. Oceanogr..

[67] Mieog J.C., Olsen J.L., Berkelmans R., Bleuler-Martinez S.A., Willis B.L., van Oppen M.J.H. (2009). The roles and interactions of symbiont, host and environment in defining coral fitness. PLoS One.

[68] Cohen I., Dubinsky Z. (2015). Long term photoacclimation responses of the coral Stylophora pistillata to reciprocal deep to shallow transplantation: photosynthesis and calcification. Front. Mar. Sci..

[69] Ben-Zvi O., Tamir R., Keren N., Tchernov D., Berman-Frank I., Kolodny Y., Benaltabet T., Bavli H., Friedman M., Glanz-Idan N. (2020). Photophysiology of a mesophotic coral 3 years after transplantation to a shallow environment. Coral Reefs.

[70] Sampayo E.M., Ridgway T., Franceschinis L., Roff G., Hoegh-Guldberg O., Dove S. (2016). Coral symbioses under prolonged environmental change: living near tolerance range limits. Sci. Rep..

[71] Scheufen T., Iglesias-Prieto R., Enríquez S. (2017). Changes in the number of symbionts and Symbiodinium cell pigmentation modulate differentially coral light absorption and photosynthetic performance. Front. Mar. Sci..

[72] Bellworthy J., Fine M. (2017). Beyond peak summer temperatures, branching corals in the Gulf of Aqaba are resilient to thermal stress but sensitive to high light. Coral Reefs.

[73] Hill R., Ralph P.J. (2005). Diel and seasonal changes in fluorescence rise kinetics of three scleractinian corals. Funct. Plant Biol..

[74] Fitt W.K., Dunne R.P., Gibb S.W., Cummings D.G., Ambarsari I., Brown B.E., Warner M.E. (1999). Diurnal changes in photochemical efficiency and xanthophyll concentrations in shallow water reef corals : evidence for photoinhibition and photoprotection. Coral Reefs.

[75] Jones R.J., Hoegh-Guldberg O. (2001). Diurnal changes in the photochemical efficiency of the symbiotic dinoflagellates (Dinophyceae) of corals: photoprotection, photoinactivation and the relationship to coral bleaching. Plant Cell Environ..

[76] Moya A., Tambutté S., Tambutté E., Zoccola D., Caminiti N., Allemand D. (2006). Study of calcification during a daily cycle of the coral Stylophora pistillata: implications for ‘light-enhanced calcification’. J. Exp. Biol..

[77] Weber J.N., Deines P., White E.W., Weber P.H. (1975). Seasonal high and low density bands in reef coral skeletons. Nature.

[78] Todd P.A., Ladle R.J., Lewin-Koh N.J.I., Chou L.M. (2004). Genotype × environment interactions in transplanted clones of the massive corals Favia speciosa and Diploastrea heliopora. Mar. Ecol. Prog. Ser..

[79] Kaniewska P., Anthony K.R.N., Hoegh-Guldberg O. (2008). Variation in colony geometry modulates internal light levels in branching corals, Acropora humilis and Stylophora pistillata. Mar. Biol..

[80] Kaniewska P., Sampayo E.M. (2022). Macro- and micro-scale adaptations allow distinct Stylophora pistillata-symbiodiniaceae holobionts to optimize performance across a broad light habitat. J. Phycol..

[81] Beltran-Torres A., Carricart-Ganivet J.P. (1993). Skeletal morphologic variation in montastrea cavernosa (Cnidaria: scleractinia) at isla verde coral reef, veracruz, Mexico. Rev. Biol. Trop..

[82] Todd P.A. (2008). Morphological plasticity in scleractinian corals. Biol. Rev..

[83] Vermeij M.J.A., Bak R.P.M. (2003). Species-specific population structure of closely related coral morphospecies along a depth gradient over a Caribbean reef slope. Bull. Mar. Sci..

[84] Dustan P. (1975). Growth and form in the reef-building coral Montastrea annularis. Mar. Biol..

[85] Hodgson G., Ginsberg R.N., Smith F.G.W. (1993). Proceedings of the Colloquium on Global Aspects of Coral Reefs: Heath, Hazards and History.

[86] Sturm A.B., Eckert R.J., Carreiro A.M., Voss J.D. (2022). Population genetic structure of the broadcast spawning coral, Montastraea cavernosa, demonstrates refugia potential of upper mesophotic populations in the Florida Keys. Coral Reefs.

[87] Labiosa R.G., Arrigo K.R., Genin A., Monismith S.G., Van Dijken G. (2003). The interplay between upwelling and deep convective mixing in determining the seasonal phytoplankton dynamics in the Gulf of Aqaba: evidence from SeaWiFS and MODIS. Limnol. Oceanogr..

[88] Kramer N., Eyal G., Tamir R., Loya Y. (2019). Upper mesophotic depths in the coral reefs of Eilat, Red Sea, offer suitable refuge grounds for coral settlement. Sci. Rep..

[89] Martinez S., Bellworthy J., Ferrier-Pagès C., Mass T. (2021). Selection of mesophotic habitats by *Oculina patagonica* in the Eastern Mediterranean Sea following global warming. Sci. Rep..

[90] Bellworthy J., Pardo R., Scucchia F., Zaslansky P., Goodbody-Gringley G., Mass T. (2022). Data from: physiological and morphological plasticity in Stylophora pistillata larvae after translocation between shallow and mesophotic reefs in Eilat, Israel. Github.

[91] Dishon G., Dubinsky Z., Fine M., Iluz D. (2012). Underwater light field patterns in subtropical coastal waters: a case study from the Gulf of Eilat (Aqaba). Isr. J. Plant Sci..

[92] Eyal G., Wiedenmann J., Grinblat M., D’Angelo C., Kramarsky-Winter E., Treibitz T., Ben-Zvi O., Shaked Y., Smith T.B., Harii S. (2015). Spectral diversity and regulation of coral fluorescence in a mesophotic reef habitat in the Red Sea. PLoS One.

[93] Tanvet C., Benzoni F., Peignon C., Thouzeau G., Rodolfo-Metalpa R. (2022). High coral recruitment despite coralline algal loss under extreme environmental conditions. Front. Mar. Sci..

[94] Kuffner I.B. (2001). Effects of ultraviolet (UV) radiation on larval settlement of the reef coral Pocillopora damicornis. Mar. Ecol. Prog. Ser..

[95] Birkeland C., Rowley D., Randall R.H., Gomez E., Birkeland C. (1981). Proceedings of the 4th International Coral Reef Symposium.

[96] Rogers C.S., Fitz H.C., Gilnack M., Beets J., Hardin J. (1984). Scleractinian coral recruitment patterns at Salt River submarine canyon, St. Croix, U.S. Virgin Islands. Coral Reefs.

[97] Wallace C.C. (1985). Seasonal peaks and annual fluctuations in recruitment of juvenile scleractinian corals. Mar. Ecol. Prog. Ser..

[98] Bradford M.M. (1976). A rapid and sensitive method for the quantitation of microgram quantities of protein utilizing the principle of protein-dye binding. Anal. Biochem..

[99] Bellworthy J., Spangenberg J.E., Fine M. (2019). Feeding increases the number of offspring but decreases parental investment of Red Sea coral Stylophora pistillata. Ecol. Evol..

[100] R Core Team (2020). R: A Language and Environment for Statistical Computing.

[101] Olito C., White C.R., Marshall D.J., Barneche D.R. (2017). Estimating monotonic rates from biological data using local linear regression. J. Exp. Biol..

[102] Platt T., Gallego C.L., Harrison W.G. (1980). Photoinhibition and photosynthesis in natural assemblages of marine phytoplankton. J. Mar. Res..

[103] Bellworthy J., Fine M. (2021). Warming resistant corals from the Gulf of Aqaba live close to their cold-water bleaching threshold. PeerJ.

[104] Liberman R., Fine M., Benayahu Y. (2021). Simulated climate change scenarios impact the reproduction and early life stages of a soft coral. Mar. Environ. Res..

[105] Görner W., Hentschel M.P., Müller B., Riesemeier H., Krumrey M., Ulm G., Diete W., Klein U., Frahm R. (2001). BAMline: the first hard X-ray beamline at BESSY II. Nucl. Instruments Methods Phys. Res. Sect. A Accel. Spectrometers, Detect. Assoc. Equip..

[106] Rack A., Zabler S., Müller B., Riesemeier H., Weidemann G., Lange A., Goebbels J., Hentschel M., Görner W. (2008). High resolution synchrotron-based radiography and tomography using hard X-rays at the BAMline (BESSY II). Nucl. Instruments Methods Phys. Res. Sect. A Accel. Spectrometers, Detect. Assoc. Equip..

[107] Zaslansky P., Fratzl P., Rack A., Wu M.-K., Wesselink P.R., Shemesh H. (2011). Identification of root filling interfaces by microscopy and tomography methods. Int. Endod. J..

[108] Schindelin J., Arganda-Carreras I., Frise E., Kaynig V., Longair M., Pietzsch T., Preibisch S., Rueden C., Saalfeld S., Schmid B. (2012). Fiji: an open-source platform for biological-image analysis. Nat. Methods.

[109] Otsu N. (1979). A threshold selection method from grey-level histograms. IEEE Trans Syst Man Cybern *SMC*-.

[110] Cumbo V.R., Edmunds P.J., Wall C.B., Fan T.Y. (2013). Brooded coral larvae differ in their response to high temperature and elevated pCO2 depending on the day of release. Mar. Biol..

[111] Bates D.W., Zimlichman E., Bolker B., Walker S. (2015). Fitting linear mixed-effects models using lme4. BMJ Qual. Saf..

[112] Ogle, D.H., Wheeler, P., and Dinno, A. (2021). FSA: Fisheries Stock Analysis.

[113] Kassambara, A., Kosinski, M., and Przemyslaw, B. (2021). Survminer: Drawing Survival Curves Using ‘ggplot2’.

[114] Doropoulos C., Evensen N.R., Gómez-Lemos L.A., Babcock R.C. (2017). Density-dependent coral recruitment displays divergent responses during distinct early life-history stages. R. Soc. Open Sci..

[115] Sims C.A., Sampayo E.M., Mayfield M.M., Staples T.L., Dalton S.J., Gutierrez-Isaza N., Pandolfi J.M. (2021). Janzen–Connell effects partially supported in reef-building corals: adult presence interacts with settler density to limit establishment. Oikos.

[116] Therneau, T.M. (2020). Coxme: Mixed Effects Cox Models.

[117] Wickham, H. (2016). Ggplot: Elegant Graphics for Data Analysis.

[118] Wilke, C.O. (2020). Cowplot: Streamlined Plot Theme and Plot Annotations for ‘ggplot2’.

