# Supplementary material for: Physiological and morphological plasticity in Stylophora pistillata larvae from Eilat, Israel, to shallow and mesophotic light conditions
Source: iScience. 2023 May 26;26(7):106969. doi: 10.1016/j.isci.2023.106969 (PMC10391605; doi:10.1016/j.isci.2023.106969)
Supplement: Document S1. Figures S1–S3 [file mmc1.pdf]

iScience, Volume *26*

Supplemental information

Physiological and morphological plasticity in *Stylophora pistillata* larvae from Eilat, Israel, to shallow and mesophotic light conditions

Jessica Bellworthy, Rachel Pardo, Federica Scucchia, Paul Zaslansky, Gretchen Goodbody-Gringley, and Tali Mass

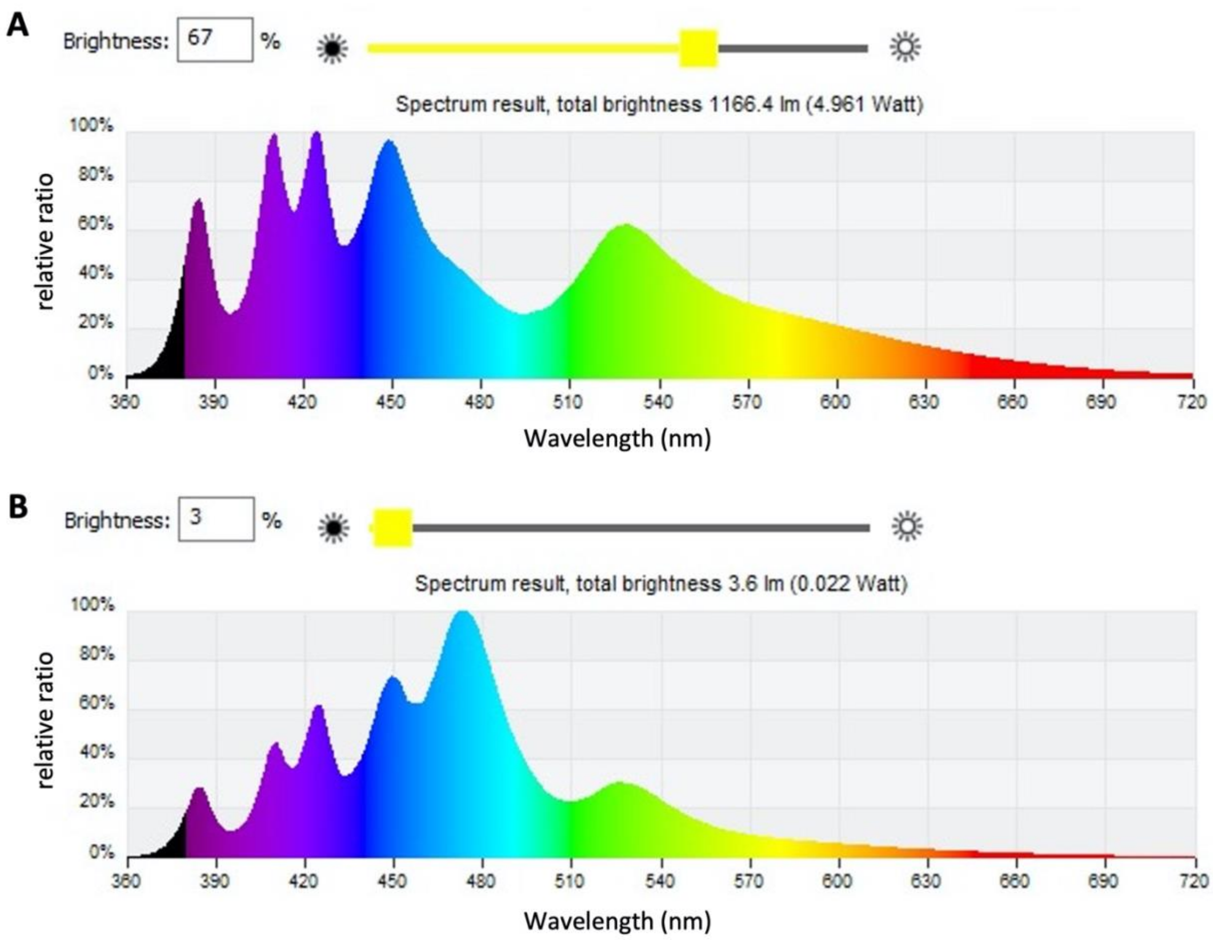

Figure S1 Spectral distribution of aquaria lights representing 'shallow' (A) and 'deep' (B) reef conditions (see also Methods Details). Each of 9 LED's, each emitting at a different wavelength, was individually set to produce the relative contribution (ratio) of wavelengths to the overall spectrum. For shallow light conditions, brightness of the lights was reduced to 67%. For deep light conditions, LED brightness was reduced to 3% to reflect differences in *in situ* light intensity at these different reef zones.

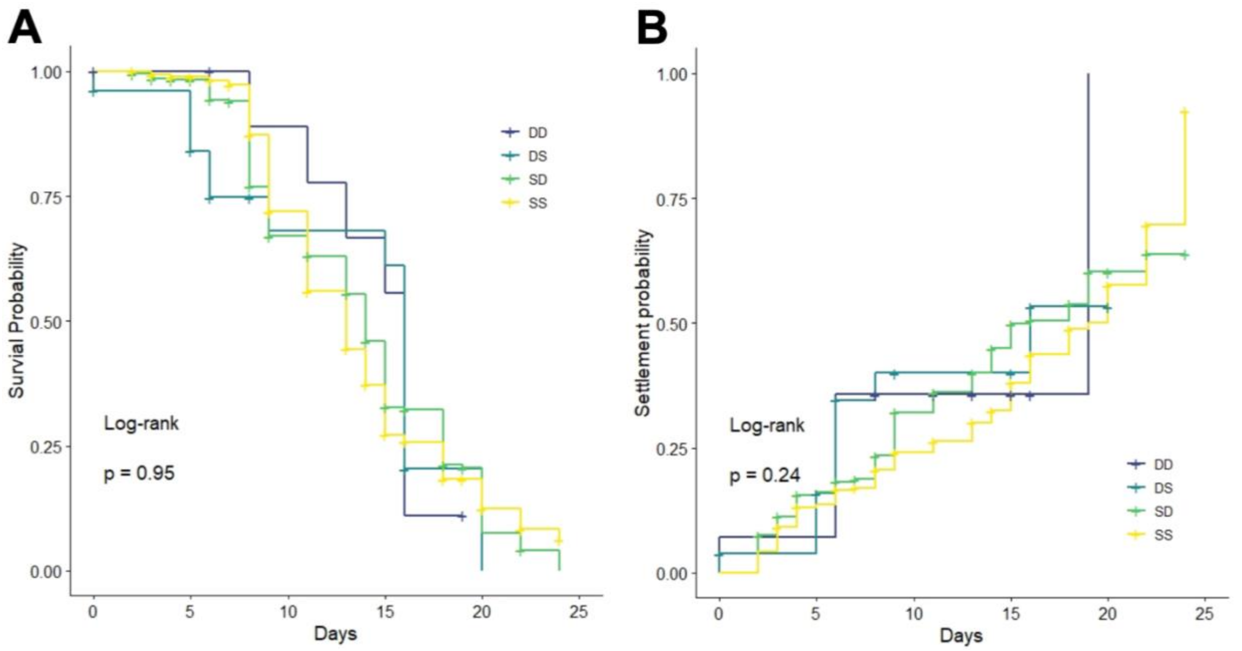

Figure S2 Kaplan Meier survival (A) and settlement (B) probability curves for coral planulae from 45 meters deep under experimental light conditions simulating the deep (DD, n = 19) or shallow reef (DS, n = 20) and planulae from 5 meters deep under experimental light conditions simulating the deep (SD, n = 146) or shallow reef (SS, n = 146). The horizontal axis is time post collection in days. A log rank test used to identify statistical differences between treatments was not significant for neither settlement nor mortality (p = >0.05).

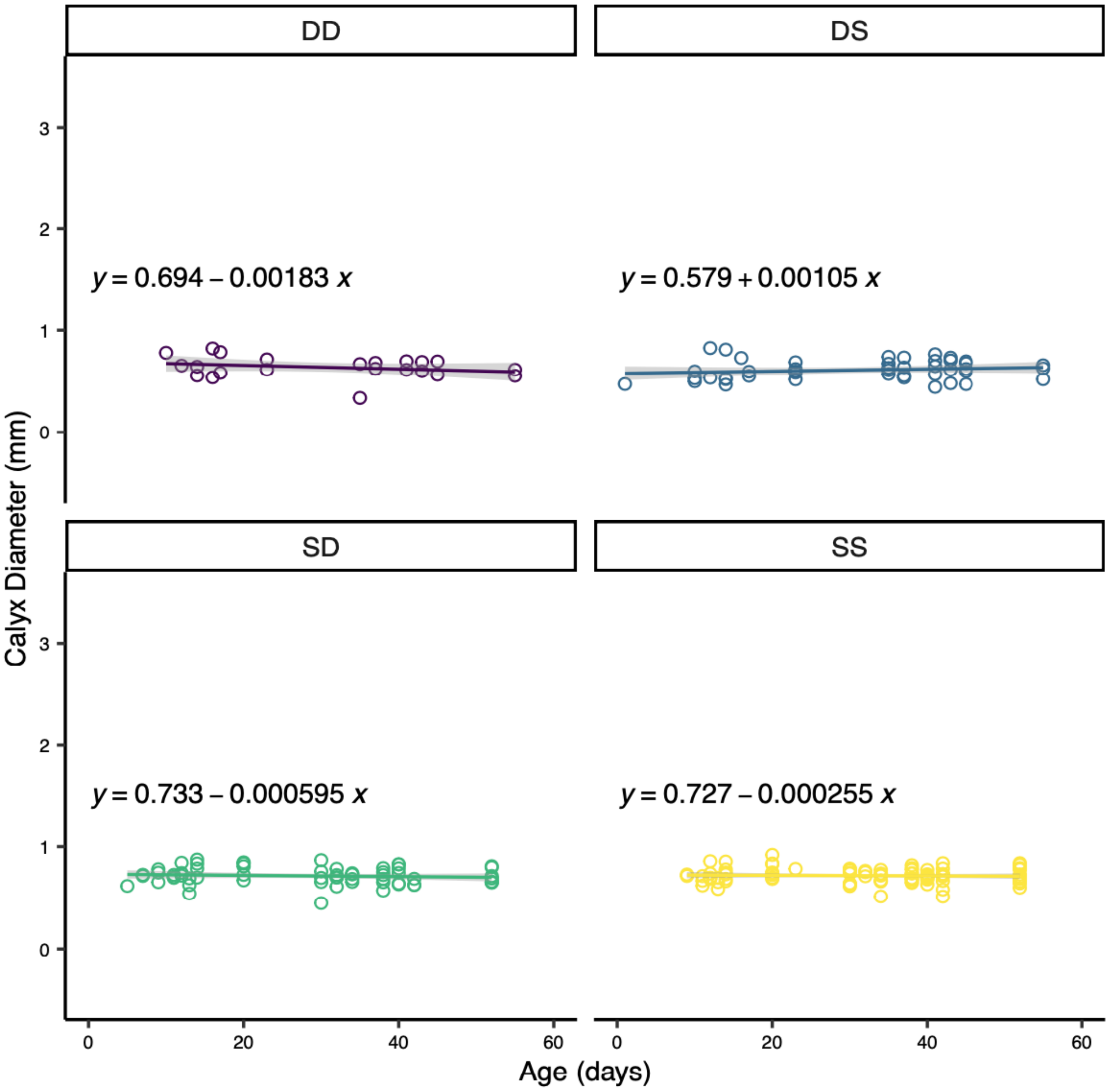

Figure S3 Measured inner calyx diameter of spat in an *ex situ* experiment (see also Fig. 3 & 4). Points are calyx diameter of individual spat with increasing age on the x-axis fitted to a linear trend line corresponding to the equation shown in each facet (n individuals per treatment: DD = 4, DS = 6, SD = 10, SS = 14). Facets represent different experimental treatments of coral planulae from 5 meters deep under high light (SS) and low light (SD) and planulae from 45 meters depth under high light (DS) or low light (DD). Grey area around the trend line is 95% confidence interval. The trend line slopes were not significantly different from zero, indicating no change in size with increasing age.
